# Gel Properties and Formation Mechanism of Camellia Oil Body-Based Oleogel Improved by Camellia Saponin

**DOI:** 10.3390/gels8080499

**Published:** 2022-08-11

**Authors:** Jing Liu, Lili Hu, Yaqing Xiao, Yingnan Liu, Songnan Li, Mingming Zheng, Zhenyu Yu, Kang Liu, Yibin Zhou

**Affiliations:** 1 Anhui Engineering Laboratory for Agro-Products Processing, Food Processing Research Institute, School of Tea and Food Science & Technology, Anhui Agricultural University, Hefei 230036, China; 2 Department of Grain Engineering, Anhui Vocational College of Grain Engineering, Hefei 230011, China; 3 Joint International Research Laboratory of Agriculture and Agri-Product Safety, Institutes of Agricultural Science and Technology Development, Yangzhou University, Yangzhou 225009, China

**Keywords:** oleogel, camellia oil body, camellia saponin, composite interfaces, mechanical property, rheological properties

## Abstract

This study aimed to investigate the effect of camellia saponin (CS) on the structural characteristics, texture properties, rheological properties, and thermal stability of camellia oil body-based oleogel (COBO). In addition, the formation mechanism of COBO was further studied in terms of the microstructure and texture of freeze-dried products, the mobility of hydrogen protons, and the conformation and structure changes of oleosin. The texture and rheological properties of the oleogels were found to be gradually improved with the incorporation of CS. This was attributed to the CS-induced enhancement of oil body interfacial film. CS was likely to bind to oleosin via hydrogen bonding and hydrophobic interactions, thereby forming a thick CS-oleosin complex interface, which was revealed by the oleosin fluorescence quenching and an increase in the ordered structure (α-helix). The composite interface could resist the crystallization damage and air disturbance caused by solidification and sublimation of water during freeze-drying, resulting in a denser and more uniform three-dimensional gel structure to trap the liquid oil, which could be explained by the decreased mobility of hydrogen protons in oleogel. The work offers a new proposal and theoretical basis for the development of saponin-enhanced oleogels using non-thermal processing.

## 1. Introduction

Oleogel, also known as oil gel, is a semi-solid oil that traps liquid oil in a three-dimensional gel network formed by gelators and stabilizers, with good texture and processing properties [1]. Moreover, it can be further modified by incorporating antioxidants to improve its oxidative stability [2]. Therefore, it is considered a promising structured oil and is favored by researchers in various fields [1,3,4,5]. In the food industry, oleogel presents an ideal green alternative to traditional solid fats in bakery, meat, and ice cream production, by exhibiting functional properties comparable to that of saturated fats, reducing fat migration rates, eliminating the harms of saturated and trans fatty acids, and promoting food qualities [6,7]. Furthermore, different oleogel-derived structures have been designed to improve the solubility of lipophilic compounds [8], modulate the release rate of free fatty acids [9], and improve the oxidative stability of bioactive compounds [10]. 

The most common method for oil gelation involves dispersing the high-melting gelator directly into oil at temperature above their melting points and then cooling to make the gelator crystallize or self-assemble to form a network structure that traps the oil [11]. The gelators used are lipophilic, including crystal particles such as glycerol monosterate [12], wax [13,14], lecithin and stearic acid [15], polymeric strands (ethyl cellulose) [16], and self-assembled low molecular weight gelator compounds such as sucrose esters [17]. The oleogel obtained displays great hardness and thermal reversibility. However, the high temperature (80 °C or higher) involved is not only harmful to the thermal sensitive components in oil but also causes oil oxidation [12,13,14,16,17,18]. Additionally, high levels (20 wt%) of gelators are required [12,15]. Emulsion templated approach is an indirect method that involves the formation and stabilization of oil-in-water (O/W) emulsion templates, enhancement of adsorption interface through cross-linking or complex formation, removal of the aqueous phase, and shearing of the dried products [19]. Since the conditions of the whole process are mild, it is friendly to heat-sensitive components in oil. Hydrophilic gelators, like polysaccharides (κ-carrageenan and pectin) [20,21], amphiphilic proteins [22], and fiber particles (plant fibers, cellulose, and hydroxypropyl methyl cellulose) [23,24] have been extensively studied for the construction of emulsion templates. However, stable artificial emulsions need to be prepared by high-pressure homogenization or high shear, which are energy-intensive processes. Up to now, there has been little literature on the development of oleogel using natural oil body emulsion templates.

Oil body is a subcellular organelle that stores triglycerides (TAGs) in a lipophilic core surrounded by a phospholipid monolayer coated with hydrophilic oleosins [25]. This special structure of the oil body makes it relatively stable in aqueous systems and is often used to build natural O/W emulsions [26]. Camellia saponin (CS), a natural surfactant from camellia plants, consists of both hydrophilic glycosides and hydrophobic pentacyclic triterpenoid ligand [27]. Due to electrostatic and/or hydrophobic interactions, CS can adsorb at the protein surface, resulting in protein-CS composite interface, thus improving emulsion stability [28]. Furthermore, CS can even form saponin-protein composite particles to enhance the stability and bio-accessibility of fat-soluble active compounds [29]. According to our preliminary study, CS bond to oleosin on the oil body surface, thus enhancing the interfacial strength of oil body film and improving the emulsion stability. Therefore, we hypothesized that CS-incorporated oil body emulsion could be a good emulsion template for the development of oleogel, avoiding the high-pressure homogenization, as the oil droplets were already wrapped in a thick interfacial film. To the best of our knowledge, the study of constructing oleogel from natural camellia oil body emulsion template (COBET) by adding CS has not been reported, and the formation mechanism is not clear.

In this study, we aimed to investigate the incorporation of CS on the texture properties, rheological properties, and thermal stability of the oleogels. In addition, the formation mechanism of camellia oil body-based oleogel (COBO) was further studied in terms of the microstructure and texture of freeze-dried products, mobility of hydrogen protons in oleogels and the conformation and structure changes of oleosin. This study can inspire the structuring of oleogel products using oil body emulsion templates strengthened with natural saponin.

## 2. Results and Discussion

### 2.1. Formation of COBO

**Figure 1:**
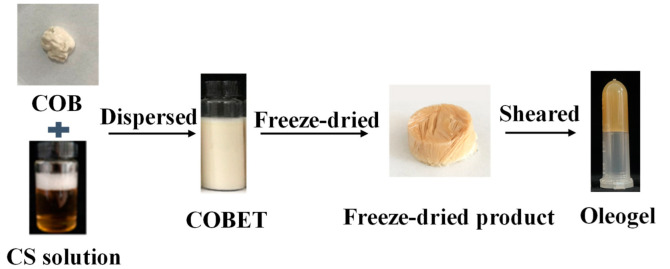
The process flow diagram for producing oleogels using COBET.

**Figure 2:**
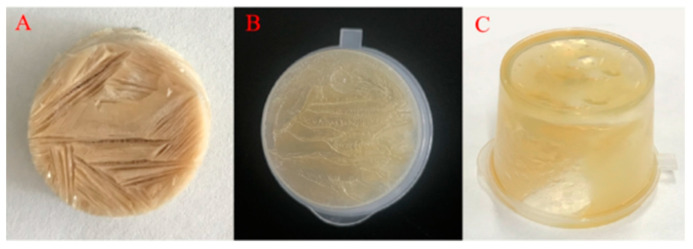
Appearance of freeze-dried products prepared with COBET containing 1.0% (**A**), 0.1% CS (**B**) and without CS (**C**).

The emulsion templated method is mainly based on the self-assembly of polymers or polymer chains to form a strong gas/oil-water interface which can remain stable during the dehydration process, thereby immobilizing oil molecules [19]. Therefore, the key to oleogel preparation through this approach is to construct a stable gas/oil-water interface that can resist external stress. Hydrophilic oleogelators, such as cellulose derivatives, protein, pectin, and xanthan gum are often used to prepare the emulsion templates [21,22,24]. For example, Meng et al. [30] prepared an oil gel using an emulsion template, in which hydroxypropyl methylcellulose was adsorbed on the oil droplets surface to stabilize the oil-water interface, while xanthan gum improved the stability of the emulsion by increasing the viscosity of the water phase. Luo et al. [21] structured an oleogel using emulsion templated method based on the orientation of tea polyphenol-palmitate particles at the oil-water interface to stabilize oil droplets and the hydration of citrus pectin to form a continuous network structure. Oil body is a natural oil storage unit wrapped by phospholipid-protein membrane. It can be extracted by the water replacement method, effectively retaining beneficial components in oil, such as vitamin E and squalene. A relatively stable natural emulsion can be obtained by simply dispersing the oil bodies in water and shearing at low speed [12]. Based on our preliminary work, the addition of CS was found to improve the stability of oil body emulsions, especially at CS concentrations greater than 0.5%. This was mainly attributed to the hydrophobic binding of CS to oleosin and the formation of composite interfaces [30], which may protect oil bodies’ integrity against external stress, such as dehydration process. Therefore, in this study, CS-incorporated COBET were first produced, as shown in Figure 1. Then the emulsion templates were freeze-dried to remove the water, resulting in soft solids with high oil contents, which was named as freeze-dried products. Finally, the freeze-dried products were sheared at low speed and a semi-solid oleogel was formed. As shown in Figure 2B, at a CS concentration of 0.1%, the freeze-dried product was gelatinous and could not be demolded, indicating insufficient CS-bind on oil body interface. However, at higher CS concentrations (1.0%), a porous and fluffy soft network was formed, and no significant oil leakage was recognized (Figure 2A). Therefore, the results supported the feasibility of preparing oleogels using COBET combined with CS.

### 2.2. Microstructure and Texture of Freeze-Dried Products

**Figure 3:**
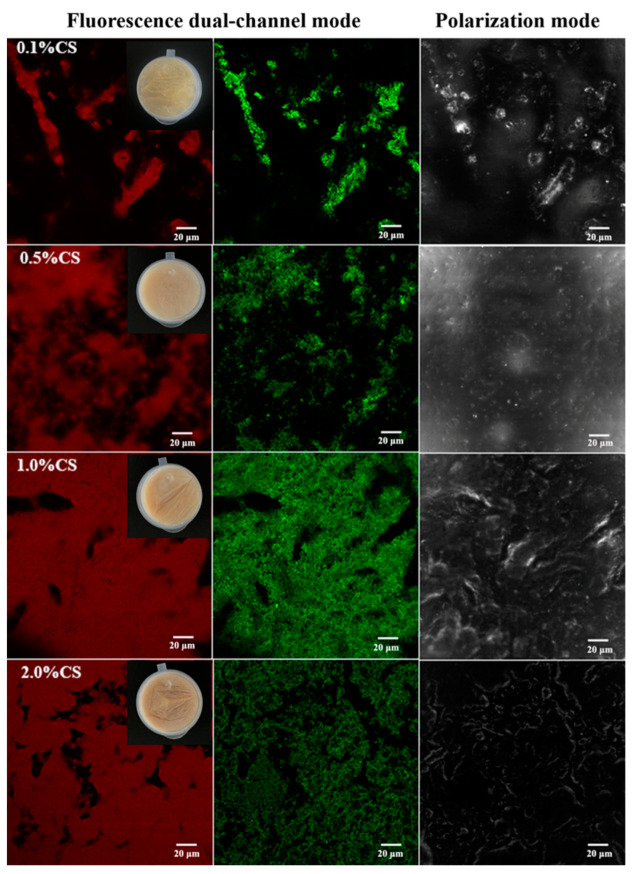
Confocal laser scanning microscope (CLSM) images of freeze-dried products from COBET with different CS content using fluorescence dual-channel mode and polarization mode. The lipid was labeled with Nile Red (in red) and the proteins was labeled with Nile Blue (in green). 0.1%CS, 0.5%CS, 1.0%CS, and 2.0%CS mean freeze-dried products from COBET containing 0.1%, 0.5%, 1.0%, 2.0% CS, respectively.

The microstructure of the freeze-dried products was evaluated by confocal laser scanning microscope (CLSM), reflecting the arrangement of oil droplets and the morphology of the oil droplet interface after freeze-drying. A loose and porous network structure, resembling a honeycomb, was identified in which oil (marked in red) was covered by a protein layer (marked in green), indicating that oil droplets were encapsulated in a network stabilized by the protein film. In addition, the higher the percentage of CS, the smaller and more uniform the pore of the network structure (Figure 3). In contrast, COBET without CS could not form an oleogel, and flowing oil was observed (Figure 2C), indicating that the oil body interface without CS adsorption was insufficient to withstand freeze-drying conditions. This fully demonstrated the contribution of CS in enhancing the strength of the oil body interfacial film, thus improving the latter’s ability to resist external stress. The polarized light microscopy (PLM) images also showed strong birefringence (Figure 3), probably due to the oriented arrangement of CS at the oil body surface, inducing crosslinking between oil bodies and supporting the formation of gel network. Zhang, Tian, Yi, Zhu, Decker and McClements [28] previously reported similar saponin-protein mixed adsorption layers at the oil-water interface. Interestingly, these layers could endure higher deformations and prevent the rupture of the oil body interfacial film [31] against external stress. 

**Figure 4:**
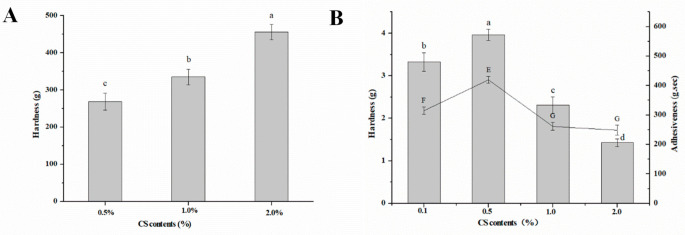
Texture properties of freeze-dried products (**A**) and oleogels (**B**) from COBET with different CS content, where the histogram (left) refers to hardness, and the line graph (right) refers to adhesiveness. Different letters (a–d) indicate significant differences (*p* < 0.05) in hardness while letters (E–G) indicate significant differences (*p* < 0.05) in Adhesiveness.

To further verify the effect of CS on the gel structure of the freeze-dried products, the hardness tests were performed, and the results were shown in Figure 4A. The addition of CS significantly increased the hardness of the freeze-dried products, indicating an enhanced network structure. It was speculated that CS was adsorbed on the oleosin surface to form an elastic multilayer film, which could resist external stress in the freeze-drying process and maintain the integrity of the oil body film, thus facilitating the formation of a denser network structure and improving the strength of the gel system.

### 2.3. Characterization of COBO

#### 2.3.1. Appearance and Microstructure of COBO

COBO was obtained by simple shearing of the freeze-dried products. The oleogels obtained were soft gels with low hardness and good adhesiveness (Figure 4B). Moreover, the gel with 0.5% CS content exhibited the best textural properties. The possible reasons are as follows: when the CS content was lower than 0.5%, there was no enough CS molecules to fully bind with oleosin and form a dense composite interface, resulting in the rupture of the oil body film during freeze-drying (Figure 2C); The oil content of COBO with 0.5% CS was 94.5% ± 0.5, which was significantly higher than that of COBO with 1.0% and 2.0% CS (92.8% ± 0.3 and 88.2% ± 0.4, respectively), thus it was speculated that the high viscosity of oil droplets limits their sliding. 

**Figure 5:**
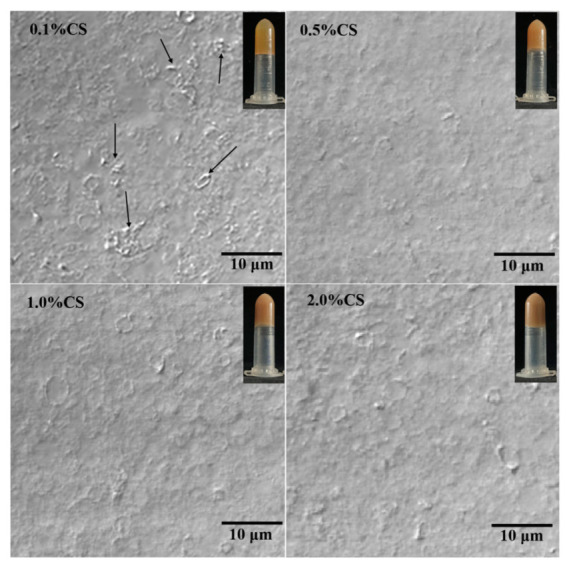
Appearance and microstructure of oleogels from COBET with different CS content under CLSM using bright-field imaging mode.

The appearance and microstructure of COBO from COBET containing different CS content are depicted in Figure 5. The oleogels from COBET with different CS content were yellow to light brown and showed immobility after inverting of centrifuge tubes. In fact, a series of polygonal pits of different sizes were recognized in the COBO micrograph, indicating droplet clusters that were tightly packed together. This was very similar to that of the oleogel reported by Tavernier et al. [19], who used protein-carrageenan composite to stabilize the interface. During the freeze-drying process, as the water was removed, the oil droplets aggregated and were rearranged. Although the emulsion droplets were clearly deformed from spherical to polygonal during drying, the interfacial film remained almost intact, indicating that the stable composite interface was the main reason for the formation of the oil-gel structure. When comparing gels with different CS content, it was found that at a CS concentration of 0.1%, the oil body film was partially ruptured, and strip-shaped protein aggregates were recognized, though most of the individual droplets were still identifiable. However, increasing the CS concentration helped to maintain the integrity of the oil body film, especially at CS concentrations of 1.0% and 2.0%. The results confirmed that CS was indeed involved in building composite interfaces on oil droplets surface and improved the stability of the interfacial films, which may further affect the texture and functional properties of oil gels. This behavior was clearly different from that of wax-based oil gels. In wax-based oil gels, the oil was trapped in a three-dimensional network structure due to weak interactions between the gelling agent and the oil, where the size, morphology, and number of wax crystal units in the oil gel determined the appearance and functionality of the oleogel [32]. 

#### 2.3.2. Rheological Properties of COBO

**Figure 6:**
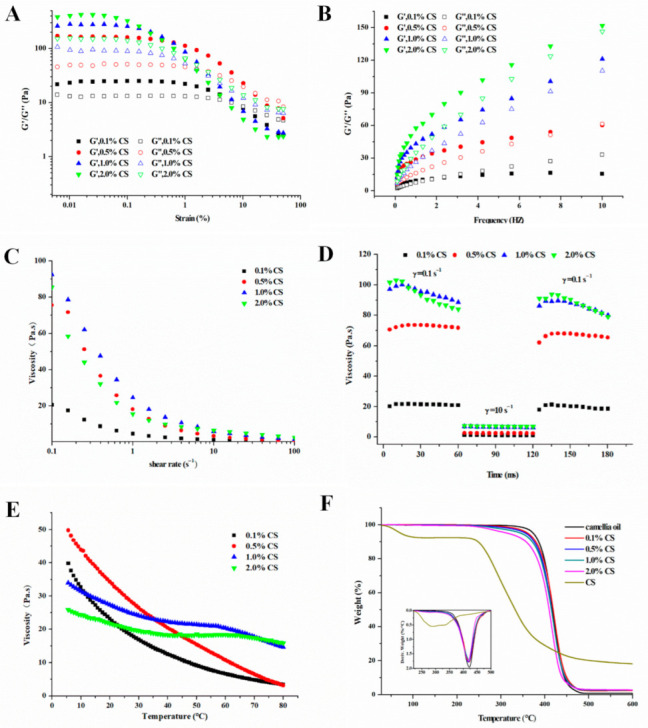
Strain sweeps (**A**), frequency sweeps (**B**), viscosity curve (**C**), thixotropic recovery curves (**D**), and temperature sweeps (**E**) and TG curves (**F**) of oleogels from COBET with different CS content.

To understand the rheological properties of the oleogels, they were characterized by oscillatory and flow sweep tests. All samples displayed shear-thinning behaviors since their viscosity declined gradually with the increasing shear stress (Figure 6A,C). This behavior was similar to that of wax-based oil gels [13]. As shown in Figure 6B, the rise in CS concentrations led to a steady rise of G’ and G″ values in linear viscoelastic region (the strain was lower than 0.1%), reflecting the formation of a more organized gel structure. It is worth noting that the G’ and G″ of the oleogels with 0.1% and 0.5% CS contents crossed at 2 Hz and 9 Hz, respectively (Figure 6B). This observation indicated the frequency-dependent attenuation and the transition from elastic to viscous responses (gel-sol transition) and could be ascribed to structural changes in the gel under continuous shearing, leading to molecular alignment and enhanced gel fluidity [24]. However, COBO containing higher CS content (1.0% and 2.0%) exhibited frequency independence, which confirmed that the incorporation of CS improved the strength and elasticity of the oleogels. This phenomenon could be attributed to the CS-oleosin interactions which improved the stability of oil body emulsion as well as the resistance of the oil body interface film to external stress during freeze-drying and shearing [28]. 

The thixotropic recovery of COBO with different CS concentrations was shown in Figure 6D. The viscosity of all COBO samples decreased slightly with time at a steady shear rate (0.1 s^−1^). However, at a higher shear rate (10 s^−1^), their structures were ruptured, causing a sharp decrease in viscosity. Nevertheless, the oleogels rapidly recovered their viscosity as the shear rate was again reduced to 0.1 s^−1^. When the CS content was 1.0%, the recovery percentage (the percentage of peak viscosity at stage 3 to that of at stage 1) of COBO decreased significantly, which was consistent with the results of textural properties. However, the thixotropic recovery of all samples was higher than 85%, showing satisfactory thixotropic recovery [1]. The results implied that the gels could potentially be applied in fields where a reversible destruction and recovery of structures are expected. 

#### 2.3.3. Thermal Stability of COBO

To evaluate the thermal stability of COBO from COBET with different CS concentrations, the temperature sweeps was performed first. As shown in Figure 6E, the viscosity of all COBO gradually decreased when the temperature increased from 5 °C to 80 °C, suggesting the fracture of oil-gel structure or the rearrangement of the oil droplets during heating and shearing. Besides, the addition of CS was found to slow down this trend, possibly because the incorporation of CS enhanced the gel network strength and increased the physical barrier between oil droplets (Figure 5), thus making the oleogels more resistant to shear stress. Combined with its low viscosity properties (Figure 6C), the oil gel may be an alternative to solid fats, such as margarine, in ice cream processing. 

Considering their application in food processing (such as baking and frying) or material making (such as edible films), the thermogravimetric (TG) curves of COBO samples were also recorded, as shown in Figure 6, F. Compared with camellia oil, the maximum decomposition temperature of CS-incorporated oleogels reduced from 421.4 °C to 417.6 °C. On the other hand, the weight loss peak of CS was at 292.8 °C. These results indicated that the addition of CS reduced the thermal cracking temperature of the oil gel. It is worth mentioning, however, that the decomposition temperatures of all CS-incorporated oleogels were much higher than that normally applied during food processing (<230 °C). In addition, all samples displayed only one cracking peak, which inferred that the CS was uniformly dispersed within the oil-gel system [32]. 

#### 2.3.4. LF-NMR of COBO

**Figure 7:**
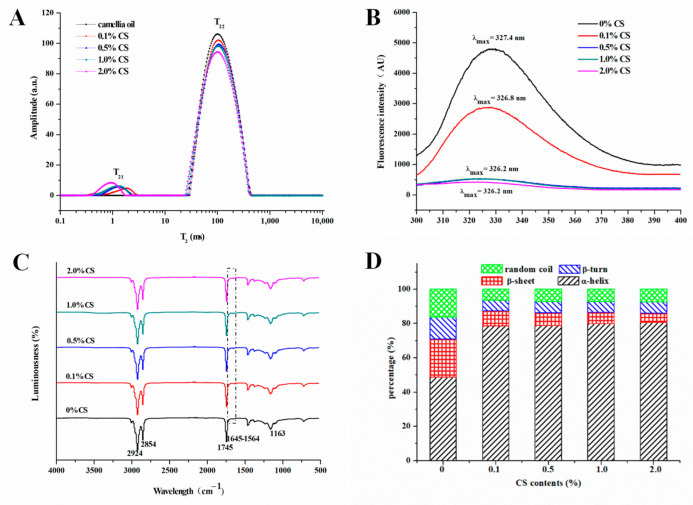
Distribution of transverse relaxation time spectra (**A**), Front-face fluorescence spectra (**B**), FTIR spectra (**C**), and secondary structure fractions of oleogels from COBET with different CS content (**D**).

LF-NMR is an effective method to evaluate the distribution and mobility of hydrogen protons in gel structures [33]. The T_2_ spectra of COBO at different CS contents was depicted in Figure 7A. Two relaxation peaks, representing protons in the residual bound water (T_21_) after freeze-drying and that of camellia oil (T_22_), were observed for all samples [34]. Among them, most of the signal came from oil molecules, indicating that most water was removed during freeze-drying and oil molecules were detained in the gel structure. In addition, as the CS percentage increased, the T_21_ relaxation peak shifted towards left, while RC_21_ increased (Table 1). This observation pointed out that more water molecules were trapped as bound water. Indeed, the addition of CS resulted in conformational changes of the oleosin (blue-shift of the fluorescence spectrum, shown in Figure 7B), exposing more hydrophilic groups and enhancing the binding capacity for water molecules [35]. Moreover, the glycosides of CS could also bind water molecules. Similarly, the T_22_ relaxation time also displayed a shift towards lower values with increasing CS concentrations, although the shift was not obvious. This implied that oil droplets were firmly confined within the cross-linked gel structure and their mobility was restricted [36]. Finally, RC_22_ was found to decrease slightly from 0.9702 to 0.9658 due to a reduction in the relative content of oil in the system (Table 1).

### 2.4. Spectral Characteristics of COBO

Front-face fluorescence spectroscopy (FFFS) has been widely employed to evaluate the micro-environment of aromatic amino acid residues in proteins by comparing fluorescence intensity and maximum wavelength (λ_max_). In Figure 7B, the FFFS of oleogels displayed a slight blue-shift with increasing CS, thereby indicating that the aromatic amino acid residues shifted to a more hydrophobic environment. Moreover, the fluorescence intensity also decreased sharply with the CS concentrations, suggesting that the adsorption of CS on the oleosin surface shielded part of the fluorescence signal [37].

The FTIR spectra of oleogels was illustrated in Figure 7C. The strong peak at 1745 cm^−1^ represented the carbonyl group of the triglycerides in oil [38]. The peaks at 1654 and 1465 cm^−1^ were ascribed to the C=O stretching and N–H bending vibration of protein amides, respectively, while those at 2924 and 2854 cm^−1^ represented the –CH_2_ stretching vibration vibrations. Finally, the peak at 1163 cm^−1^ was mainly attributed to the C–O stretching [38]. With the addition of CS, there was no significant change in the position of the peaks, indicating that no new chemical group was generated. These results supported the idea that a CS-oleosin complex structure could be formed through non-covalent interactions. The secondary structure fractions of COBO with different CS content were also depicted in Figure 7D. The incorporation of CS caused a marked increase in the α-helix structure of oleosin, along with a dramatic decrease in other structures (β-sheet, β-turn and Random coil). This was an indication of structure changes in oleosin as well as the formation of elastic oil-water multilayer interfaces, as α-helix structures were always associated with elastic multilayer interfaces, whereas β-sheet structures often reflected a rigid protein network [39].

### 2.5. The Mechanism of COBO Formation

**Figure 8:**
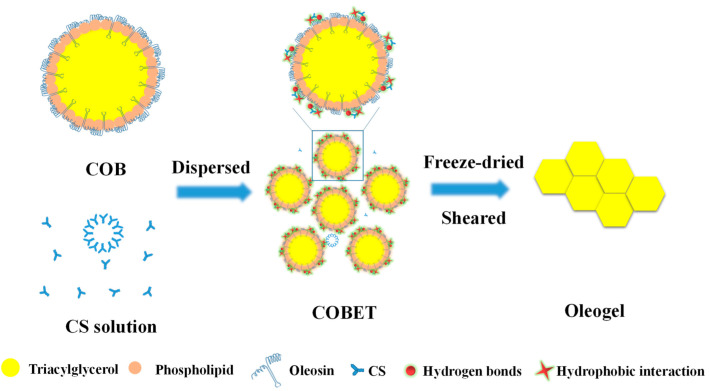
The possible mechanism for the development of oleogels using COBET incorporated with CS.

The possible mechanism of oleogel formation using CS-incorporated camellia oil body emulsion templates was proposed, as shown in Figure 8. It is already established that oleosins, embedded in phospholipid monolayer, are major contributors to the interfacial properties of oil bodies. The hydrophobic central domain in the oleosin molecule forms an 11 nm stalk-like structure, extending inwardly into the interior of the triacylglycerol and the hydrophobic acyl interior of the phospholipid. The amphiphilic NH_2_-terminal and COOH-terminal domains of oleosin, on the other hand, cover the surface of the oil body and provide steric hindrance as well as electrostatic repulsion to maintain the stability of the oil bodies [40]. Moreover, the α-helix structure at the COOH-terminal may also increase the hydrophobicity of oleosin, increasing the stability of the oil body structure through hydrophobic interactions [25]. Altogether, these features allow natural oil bodies to be dispersed in aqueous solutions to form O/W emulsions and resist high-speed centrifugation. However, the monolayer phospholipid membrane is rigid and poorly elastic. As such, not only can it be punctured by ice crystals formed during freezing, but it also cannot withstand the air disturbance caused by sublimation of ice crystals during freeze-drying, resulting in oil leakage (Figure 2C). Interestingly, the hydrophilic glycosides of CS are rich in hydroxyl groups, making it likely to interact with the hydrophilic COOH– and NH_2_–termini of oleosin via hydrogen bonding, as revealed by the increase in α-helix structure of oleosin (Figure 7D) [41]. Meanwhile, due to the hydrophobicity of the α-helix structure in the COOH–termini of oleosin, it is possible to hydrophobically bind to the pentacyclic triterpenoid ligand of CS. As a result, an elastic CS-oleosin complex interfacial film can be formed through hydrogen bonds and hydrophobic interactions, as also evidenced by the protein fluorescence quenching of oleogel (Figure 7B) [37]. During freezing, the coated oil droplets are aligned along the direction in which the ice crystals are formed. During the subsequent dehydration process, the encapsulated oil droplets aggregate and cross-link to form a continuous gel structure and restrict the mobility of oil molecules. 

## 3. Conclusions

This study investigated the influence of CS on the gel properties of COBO and explored the formation mechanism. The results demonstrated that continuously increasing the amount of CS could improve the pore uniformity and hardness of the freeze-dried products, thereby influencing the texture properties, rheological properties, and thermal stability of the COBO. This was due to the formation of the elastic composite interface on the oil body surface as CS bound to oleosin through hydrogen bonds and hydrophobic interactions, as indicated by the decrease in oleosin fluorescence intensity and the increase in α-helix structures. The composite interface could effectively resist the crystallization damage and air disturbance caused by solidification and sublimation of water during freeze-drying, thereby promoting the formation of a three-dimensional gel structure, which immobilizes liquid oils, as reflected by the reduction in hydrogen protons mobility in oleogel. This result provided the potential utilization of camellia oil bodies emulsion combined with CS as the templates for the preparation of oleogel. Future research may also involve studies of the oxidative stability and digestive properties of oleogels, aiming to produce products with excellent functional and nutritional properties.

## 4. Materials and Methods

### 4.1. Materials

Camellia seeds were obtained from Anhui Longcheng Biological Technology Co. LTD at harvest and stored at 4 °C; while CS, purchased from Tong Ze Bio-technology Co., Ltd. (Shanxi, China), contained saponins (95.0%), proteins (0.5%), ash (2.7%) and water (2.8%) determined experimentally. Nile Red and Nile Blue were purchased from Aladdin Reagent (Shanghai, China). De-ionized water was obtained with a Merck Millipore device (Darmstadt, Germany) and used for all media preparation. 

### 4.2. Isolation and Purification of Camellia Oil Body (COB)

COB was extracted and purified as described by Ding, Xu, Qi, Jiang and Sui [42] with some modifications. Camellia seeds were washed and soaked in deionized water at 4 °C overnight. Then they were ground in 0.1 M sodium chloride solution (1:10 (*w*/*v*); pH 7.0, 4 °C) using a beater (MJ-60BE01B, Midea, Foshan, China) at full power for 180 s. The resulting slurry was filtered through three layers of gauze prior to centrifugation of the filtrate in a refrigerated centrifuge (JW3021HR, Jiawen Co., Ltd., Hefei, Chain) at 10,000× *g* and 4 °C for 30 min. The creams, rich in COB, were then isolated and dispersed in 20% of sucrose solution for 30 min in a ratio of 1:4 (*w*/*v*) using a magnetic stirrer. The pH of the dispersed sample was adjusted to 11.0 with 1 M NaOH solution and then centrifuged to isolate the cream, with this dispersal and cream collection process repeated four times. Finally, to remove any remaining sucrose in the COB, the latter was mixed with deionized water (1:8, *w*/*v*) and subsequently centrifuged (10,000× *g*, 4 °C) for 30 min. 

### 4.3. Preparation of COBO

COBO was prepared following the protocol of Tavernier, Patel, Van der Meeren, and Dewettinck [43] with some modifications. Briefly, CS was mixed with deionized water and magnetically stirred at 25 °C overnight with the final concentrations of CS in solutions being 0.1%, 0.5%, 1.0% and 2.0% (*w*/*v*). COBET were prepared by dispersing 20% (*w*/*v*) of COB in the prepared CS solutions using a high-speed homogenizer (FJ200-S, Lichen Technology Co., Ltd., Shanghai, China) at 10,000 r/min for 1 min. The pH of all samples was adjusted to 7.0 using 1 M NaOH solution. Then the samples were frozen at −20 °C for 24 h and freeze-dried in a freeze dryer (YTLG-10A, Yetuo Equipment Co., Ltd., Shanghai, China) for 36 h. The COBO was obtained by shearing the resulting freeze-dried products with the help of a high-speed homogenizer at 2000 r/min for 1 min.

### 4.4. Microstructure Observation

The microstructure of the freeze-dried products and oleogel samples were acquired on a confocal laser scanning microscope (FV3000, Olympus Corporation, Tokyo, Japan) [28]. A fluorescence dual-channel mode and a polarization mode was used for microscopic imaging of freeze-dried products, in which Nile Red (0.1%, *w*/*v*) and Nile Blue (0.1%, *w*/*v*) were used to label lipids and proteins, respectively. Prior to observation, 5 μL of pre-fluorescently stained COBET was placed into the groove of the slide and covered with a coverslip, then it was freeze-dried. A bright field imaging mode was adopted for oleogel microscopic imaging. All images were trapped with a 20× eye lens and 20× objective lens.

### 4.5. Texture Test

The texture tests were performed on a texture analyzer (TA.XT. Plus, Stable Micro systems, Surrey, UK), equipped with a 50 kg force sensing unit [24]. The hardness of freeze-dried products was determined using a P36R probe with a test speed of 1.0 mm/s, a compression distance of 5.0 mm, and a trigger force of 5.0 g. Similarly, the hardness and adhesiveness test of oleogel samples were conducted using a P0.5 probe. The pretest speed, test speed and posttest speed were 0.2 mm/s while the test distance and trigger force were 2.0 mm and 5.0 g, respectively. The oleogel samples were placed in a 24-well microtiter plate (gently vibrated to remove air) prior to the tests. 

### 4.6. Rheological Analysis of COBO

The rheological properties of COBO samples were determined on a Discovery HR-1 rheometer (TA Instruments, New Castle, DE, USA), equipped with a 40 mm diameter parallel plate for which the gap was set to 1000 μm [21]. The linear viscoelastic region of the samples was determined by strain sweep at a frequency of 1 Hz in a strain range of 0.01–50%, while frequency sweep tests were performed at frequencies from 0.1 Hz to 10 Hz and a strain of 0.04%. Thixotropic recovery tests were carried out sequentially at shear rates of 0.1 s^−1^, 10 s^−1^ and of 0.1 s^−1^ for 1 min each. All tests above were carried out at 25 °C. Thermal sensitivity was measured by temperature sweeps in the range of 5 °C to 80 °C at a rate of 2 °C/min.

### 4.7. Thermogravimetric (TG) Analysis

The TG analysis of the COBO samples was performed on a thermogravimetric analyzer (TGA/SDTA851, Mettler Toledo, Zurich, Switzerland) [44]. Oleogel samples (10.0 mg) were weighed into a porcelain crucible and heated from 25 °C to 600 °C at a rate of 10 °C/min under a N2 flow of 20 mL/min.

### 4.8. LF-NMR

An NMI20-Analyzer (Niumag Electric Corporation, Shanghai, China) was used to investigate the mobility of specific hydrogen protons in oleogel samples based on the method of [33]. Measurement was performed at a magnetic field strength of 0.53 T and a temperature of 32.0 °C using the Carr-Purcell-Meiboom-Gill sequence. The transverse relaxation time (T_2_) and the relative contribution percentage (RC_2_) of oleogel samples were recorded.

### 4.9. Spectroscopic Analysis

#### 4.9.1. Front-Face Fluorescence Spectroscopy (FFFS)

The FFFS of oleogel samples was performed using a F-7000 FL Spectrophotometer (Hitachi High-Tech, Tokyo, Japan), equipped with a front-face accessory [37]. The emission spectra were recorded in the range of 310 nm to 400 nm under an excitation wavelength of 290 nm at 25 °C. The slit widths of excitation and emission wavelengths were 5 nm. 

#### 4.9.2. FTIR Spectroscopy

The FTIR spectra for COBO samples was recorded on a Bruker Alpha FTIR spectrometer (Bruker Optics GmbH, Ettlingen, Germany) using an ATR mode in the spectral range of 4000–400 cm^−1^ with 32 scans at a resolution of 4 cm^−1^ [13]. Spectral analysis was performed using an OMNIC software (Thermo, V8.2, Madison, WI, USA). The relative content of protein secondary structures was assessed by calculating the relative peak areas of the resolved amide I region based on the second derivative amide I spectra.

### 4.10. Statistical Analysis

Measurements were carried out at least in triplicate. Data were reported as means ± standard deviations, and the significant difference (*p* < 0.05) was identified via Duncan’s tests using SPSS software. 

## Figures and Tables

**Table 1:** LF-NMR relaxation information and oil percentages of oleogels from COBET with different CS content.

CS Content (%)	T_21_ (s)	T_22_ (s)	RC_21_ (%)	RC_22_ (%)	Oil Percentages (%)
0 0.1	- 0.595 ± 0.035 ^a^	29.392 ± 0.783 ^a^ 30.366 ± 0.646 ^a^	- 0.0248 ± 0.0004 ^c^	0.9985 ± 0.0007 ^a^ 0.9752 ± 0.0004 ^b^	- 95.19 ± 0.05 ^a^
0.5	0.565 ± 0.007 ^a^	29.134 ± 0.537 ^a^	0.0293 ± 0.0008 ^b^	0.9707 ± 0.0008 ^c^	93.45 ± 0.09 ^b^
1.0	0.430 ± 0.011 ^b^	26.373 ± 0.652 ^b^	0.0298 ± 0.0010 ^b^	0.9702 ± 0.0009 ^c^	91.42 ± 0.08 ^c^
2.0	0.400 ± 0.014 ^b^	24.827 ± 0.905 ^b^	0.0392 ± 0.0004 ^a^	0.9608 ± 0.0004 ^d^	88.23 ± 0.05 ^d^

The group of 0 presents camellia oil as a control. The same superscript letters in the same column are not significantly different (*p* > 0.05) according to Duncan’s multiple range tests.

## Data Availability

Data obtained as described.
